# 2691. Does Janus Kinase Inhibitor Increase the Risk of Herpes Zoster in Rheumatoid Arthritis Patients in Saudi Arabia? A Multicenter Retrospective Cohort Study

**DOI:** 10.1093/ofid/ofad500.2302

**Published:** 2023-11-27

**Authors:** Fida Ahmed, Reham Kaki, Ola Abudaowd, Suzan Attar, Hani Almoallim, Gamal Salama

**Affiliations:** King Abdulaziz university, Department of Infectious Disease and Department of Infection Control and Environmental Health, Jeddah, Saudi Arabia, Jeddah, Makkah, Saudi Arabia; King Abdulaziz university, Department of Infectious Disease and Department of Infection Control and Environmental Health, Jeddah, Saudi Arabia, Jeddah, Makkah, Saudi Arabia; King Faisal specialist hospital and research center, Internal Medicine, Jeddah, Saudi Arabia, Jeddah, Makkah, Saudi Arabia; King Abdulaziz University Hospital, Internal medicine, division of Rheumatology, Jeddah, Saudi Arabia, Jeddah, Makkah, Saudi Arabia; Umm Alqura University, Medicine, division of Rheumatology, Makkah, Saudi Arabia, makkah, Makkah, Saudi Arabia; Al Azhar university, Rheumatology, Asir , Saudi Arabia, asir, Asir, Saudi Arabia

## Abstract

**Background:**

JAK inhibitors (JAKi) are targeted synthetic disease-modifying antirheumatic drugs. Current evidence showed they are effective in either post Conventional or Biologic disease-modifying anti-rheumatic drugs ( cDMARDs) (bDMARDs) in patients with moderate to severe rheumatoid arthritis (RA)

JAKi could impair the immune response to viral infection by interfering with interferon signaling through JAK/STAT pathway.

Emerging Data from phase II/III clinical trials, long-term follow-up, and observational studies suggested an increased risk of viral infections, especially herpes zoster (HZ) virus reactivation in patients treated with JAKi.

Our aim was to evaluate the risk of herpes zoster in rheumatoid arthritis patients receiving JAK inhibitors.

**Methods:**

A multicenter retrospective cohort study in Saudi Arabia. Rheumatoid arthritis patients aged 18 and above from the period of 2007-2022 were included.

Medical records were reviewed, and patients were phone interviewed to register an outcome of HZ infection.

Chi-square was used to assess the correlation between various risk factors and HZ infection.

**Results:**

Of a total of 308 patients, 108 were on JAKi.

JAKi didn’t significantly increase the risk of HZ development (OR1.97, 95% CI 0.71- 4.67).

Patients on Etanercept had a higher risk of HZ (OR 2.88, 95% CI 1.65-2.83).

3 out of the 7 cases who developed HZ were on chronic corticosteroids. However, it wasn’t significantly associated with HZ devolvement.

Patients with Asian ethnicity were significantly more predisposed to HZ infection (OR 1.2, 95% CI 0.8-4.37).

**Figure:**
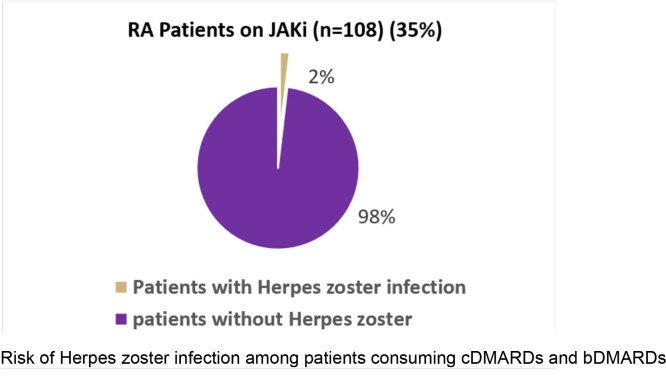

**Figure:**
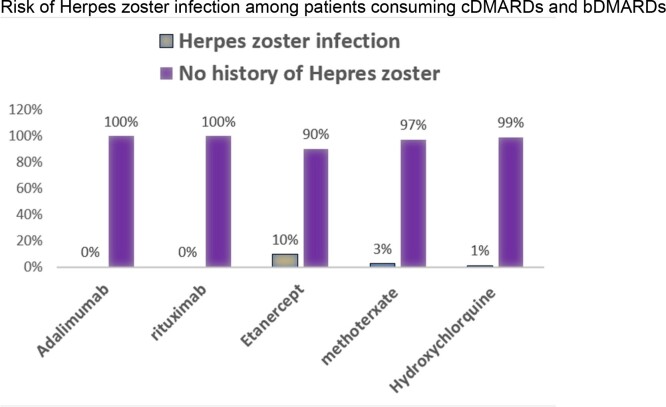

**Conclusion:**

In this multicenter study in Saudi Arabia, JAKi were not associated with the development of HZ among Rheumatoid Arthritis patients in real-world data.

As with any observational dataset cause and effect cannot be established with certainty as residual confounding may remain.

This finding would support the evaluation of zoster vaccination in RA patients.

**Disclosures:**

**All Authors**: No reported disclosures

